# Diversity Feature Learning Network for Occluded Person Re-Identification

**DOI:** 10.3390/s26165160

**Published:** 2026-08-14

**Authors:** Lei Qi, Liejun Wang, Shaochen Jiang

**Affiliations:** 1 School of Computer Science and Technology, Xinjiang University, Urumqi 830046, China; qilei@xju.edu.cn (L.Q.); scj@xju.edu.cn (S.J.); 2 Xinjiang Multimodal Intelligent Processing and Information Security Engineering Technology Research Center, Urumqi 830017, China

**Keywords:** occluded person re-identification, occlusion simulation, diversity constraint, feature fusion

## Abstract

**Highlights:**

**What are the main findings?**

- DFLNet achieves accurate pedestrian retrieval in occlusion scenarios by learning diverse semantic information about target pedestrians and effectively fusing occluded and non-occluded features.
- SLO strategy automatically simulates two common occlusion scenarios based on the relative positions of obstacles and target pedestrians in the real world.
- TDC Loss enforces the model to learn differentiated feature representations by imposing constraints on extended multiple class tokens.
- DFF module achieves the interaction and fusion of dual-branch features by establishing global correlations among features and optimizing the distribution distances between them.

**What are the implications of the main findings?**

- DFLNet effectively mitigates the interference caused by non-target features in occlusion scenarios through data augmentation strategies at the input stage, feature learning strategies at the feature stage, and feature fusion strategies at the output stage.

**Abstract:**

Occluded person re-identification (Re-ID) is a challenging task, as non-target pedestrians or surrounding obstacles often interfere with the visual cues of the target person, making it difficult for models to effectively learn discriminative feature representations. Most existing methods focus on salient body parts via spatial partitioning or external cues; however, they are either limited in capturing diverse semantic information or tend to introduce additional network complexity. To address these issues, we propose a Diversity Feature Learning Network (DFLNet). Specifically, a Scene-Level Occlusion (SLO) strategy is designed to automatically simulate two common occlusion scenarios by modeling the relative spatial relationships between the target person and surrounding occluders in real-world scenes. Subsequently, multiple class tokens are introduced to capture diverse representations of the target identity. A Token Diversity Constraint (TDC) loss is further imposed on these class tokens to encourage the learning of discriminative and diverse feature embeddings. Finally, we design a Diversity Feature Fusion (DFF) module, which facilitates the interaction and integration of dual-branch features by modeling global feature correlations and optimizing inter-feature distribution distances. Extensive experiments on occluded, partial, and holistic Re-ID datasets demonstrate the effectiveness of the proposed DFLNet.

## 1. Introduction

Person Re-identification (Re-ID) aims to accurately match the same individual across different camera views and is a key technology for enhancing public security and assisting criminal investigations [1,2,3,4]. Existing studies primarily focus on holistic person Re-ID; however, in real-world surveillance scenarios, target pedestrians are often partially occluded by vehicles, billboards, or other pedestrians, resulting in incomplete visual information and significantly degrading recognition performance. Therefore, developing a robust person Re-ID model capable of effectively handling occlusions is of both significant research value and practical importance.

Compared with holistic person Re-ID, occlusions introduce substantial irrelevant information that interferes with the extraction of discriminative features, making person re-identification under occluded scenarios considerably more challenging. In recent years, numerous methods have been proposed for occluded Re-ID, which can be broadly categorized into two groups: (1) partition-based methods and (2) external cue-based methods. Partition-based methods [5,6,7,8] typically divide feature maps into several predefined regions to learn feature representations from different body parts. However, due to the large variations in pedestrian pose, viewpoint, and spatial location, such rigid partitioning often disrupts the semantic integrity of the human body (e.g., splitting the head into multiple regions), leading to feature misalignment and suboptimal matching performance. In contrast, external cue-based methods [9,10,11,12] leverage auxiliary information, such as pose estimation or human parsing, to identify visible body regions while suppressing the influence of occluded areas. Nevertheless, these approaches heavily rely on human structural priors, which may overlook discriminative visual cues beyond body structure, such as backpacks and clothing textures. Moreover, the introduction of auxiliary models inevitably increases computational complexity and implementation cost.

To address the limitations of existing methods, we propose a Diversity Feature Learning Network (DFLNet). First, to mitigate the limited availability of occluded samples in training datasets, we introduce a Scene-Level Occlusion (SLO) strategy. By dynamically generating diverse occlusion patterns, SLO alleviates the distribution imbalance between occluded images and non-occluded images during both training and inference stages. To explicitly model the consistency of pedestrian features under occluded and non-occluded conditions, this paper adopts a dual-stream network architecture. Since occluded samples introduce a significant amount of background noise and semantic information loss, processing them together with the original samples can easily lead to difficulties in feature alignment. Therefore, we process the occluded and original samples separately and establish dedicated mapping spaces for different features. Second, multiple class tokens are introduced to enable the model to attend to different body regions of the target pedestrian, while a Token Diversity Constraint (TDC) loss is imposed to ensure that each token learns discriminative and complementary feature representations. Finally, we design a Diversity Feature Fusion (DFF) module, which facilitates interaction and fusion between the two feature branches. In addition, an angular distance constraint is incorporated to refine the embedding space, thereby reducing the distribution discrepancy between occluded and holistic feature representations.

In summary, the main contributions of this work are summarized as follows:

(a) We propose a Scene-Level Occlusion (SLO) strategy based on the relative spatial relationships between target pedestrians and occluders in real-world scenarios. This strategy simulates both pedestrian-induced occlusions and object-induced occlusions during the training phase, thereby enhancing the model’s robustness and adaptability to diverse occlusion scenarios.
(b) We introduce a Token Diversity Constraint (TDC) loss, which imposes constraints on the expanded class tokens to mitigate feature homogenization among them, thereby encouraging the model to learn more discriminative and diverse feature representations.
(c) We design a Diversity Feature Fusion (DFF) module, which enables effective interaction and fusion between dual-branch features, thereby allowing the model to more accurately focus on the same target pedestrian under both occluded and non-occluded scenarios.

## 2. Related Work

### 2.1. Occluded Person Re-Identification

Currently, methods for addressing occluded person re-identification can be broadly categorized into three groups: CNN-based methods, external cue-based methods, and Transformer-based methods.

CNN-based methods primarily address occlusion by leveraging local feature alignment. Sun et al. [13] proposed a part-based modeling approach that partitions feature maps horizontally. Specifically, the feature map is uniformly divided into multiple horizontal stripes, enabling the convolutional network to learn discriminative local representations corresponding to different body parts of pedestrian images. Chen et al. [7] introduced an attention mask module that adaptively enhances visible body regions while suppressing irrelevant or occluded regions during feature extraction. Yan et al. [5] designed an adaptive feature map partitioning strategy based on the importance of different body regions, strengthening local representations of visible parts while reducing the influence of occluded or noisy regions. Wang et al. [14] proposed an identity-aware spatial attention module combined with an adaptive global feature extraction mechanism, jointly optimizing local occlusion handling and global feature enhancement, thereby improving re-identification performance under occluded scenarios.

External cue-based methods leverage pose estimation or semantic segmentation to indicate visible human body parts. Miao et al. [15] utilized pose-estimation-based attention maps to guide the network in focusing on unoccluded body regions during feature alignment. Wang et al. [9] extracted semantic joint locations of pedestrians via keypoint estimation and employed a graph convolutional network to model the underlying human body topology. Wang et al. [16] adopted pose estimation techniques to accurately locate human keypoints and perform selective similarity computation on visible regions. Somers et al. [10] integrated pedestrian identity information with semantic labels from human parsing, and introduced a body-part attention module to guide the network in attending to different semantic regions.

Transformer-based methods leverage self-attention mechanisms to capture long-range dependencies in pedestrian images, explicitly modeling relationships among image patches through global feature interactions. Li et al. [17] combined a multi-granularity feature learning framework with a Transformer architecture to enhance the diversity of feature mining for occluded pedestrians. He et al. [18] introduced a jigsaw patch module to improve perturbation invariance of local features, and incorporated side information embedding to mitigate feature biases caused by viewpoint and camera variations. Tan et al. [19] performed cross-layer aggregation of multi-scale patch features to adaptively select high-quality global prototype representations while extracting effective local features under occlusion. Li et al. [20] proposed a second-order attention module to model higher-order semantic relationships among body parts, together with an entropy-guided feature fusion mechanism to adaptively integrate multi-scale information. Zhang et al. [21] further exploited cross-layer multi-scale patch aggregation to extract and fuse hierarchical fine-grained details, thereby strengthening the model’s discriminative capability in occluded scenarios.

### 2.2. Occlusion Simulation

Occlusions introduce substantial noise, thereby limiting the robustness of existing person re-identification models. A key reason lies in the scarcity of occluded samples in training datasets, which prevents the model from adequately learning the underlying relationships between occlusion patterns and pedestrian identity features. To address this issue, applying controllable occlusion-based data augmentation during the training stage can effectively enhance the model’s ability to recognize target pedestrians in complex occluded scenarios.

Existing occlusion simulation strategies can be broadly categorized into two types. The first type is random occlusion strategies [7,22,23,24], which typically simulate occlusions by randomly generating rectangular regions and performing pixel replacement, erasing, or pasting. These methods are simple to implement and can effectively mitigate overfitting; however, the generated occlusions often lack semantic information (i.e., non-semantic occlusions) and are therefore unable to faithfully simulate complex object occlusions in real-world scenarios, limiting the model’s generalization capability. The second type consists of occlusion synthesis methods based on cropped real occluders [25,26,27,28,29], which extract occluders from the training set either manually or automatically and synthesize them onto pedestrian images. Although these methods improve the semantic realism of the generated samples, they do not adequately consider the spatial relationships between the target pedestrian and the occluder, thereby limiting the diversity of the simulated scenarios.

Unlike the aforementioned methods, our proposed occlusion enhancement strategy deeply integrates semantic priors and spatial knowledge from real-world scenarios, enabling comprehensive simulation of diverse occlusions caused by pedestrians and objects.

## 3. Method

### 3.1. Network Architecture

**Figure 1:**
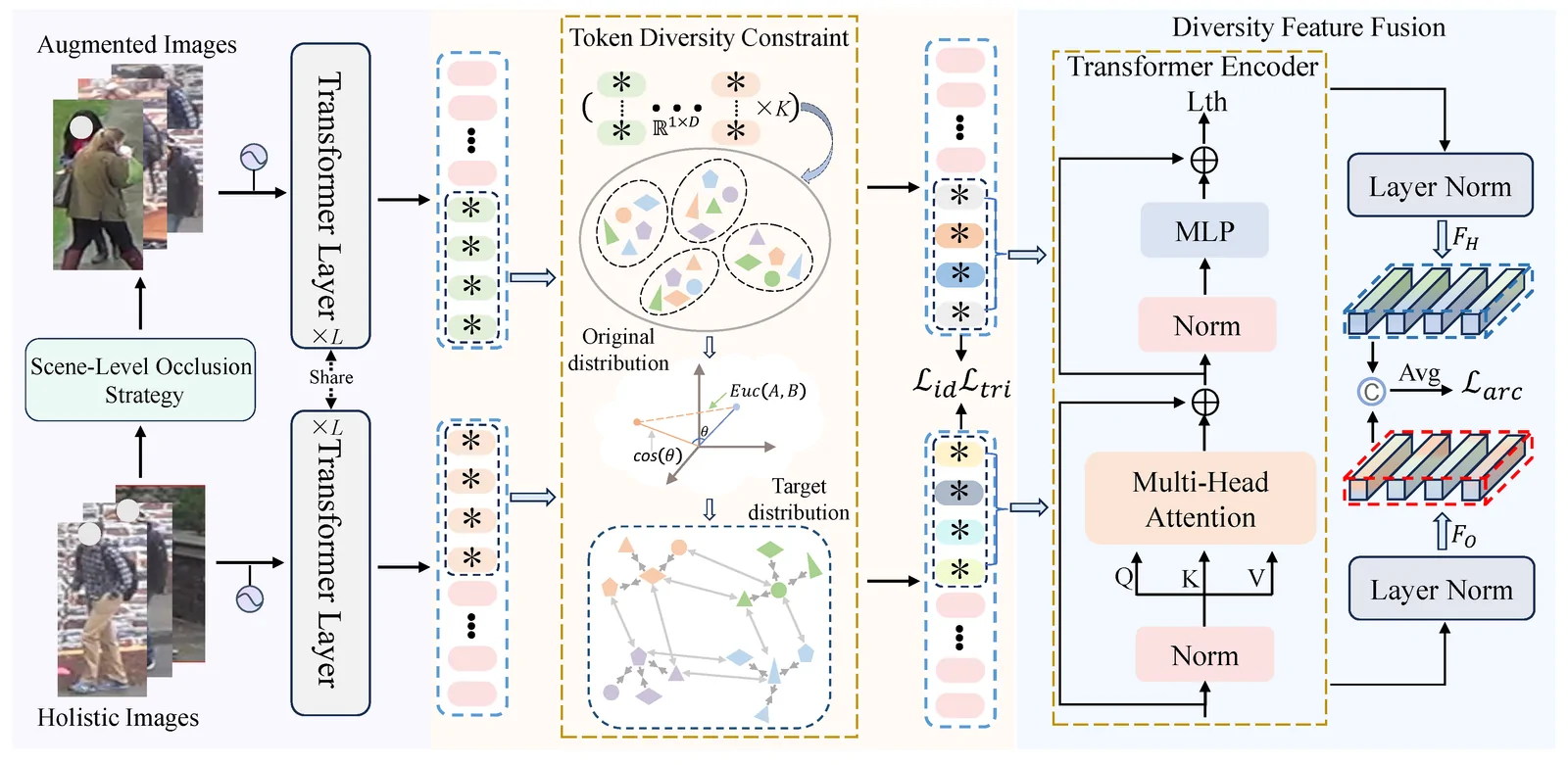
The overall framework of the Diversity Feature Learning Network (DFLNet). DFLNet consists of the Scene-Level Occlusion (SLO) Strategy, Token Diversity Constraint (TDC) Loss, and Diversity Feature Fusion (DFF) Module. The SLO strategy simulates the relative spatial relationships between target pedestrians and environmental occluders in real-world scenes, and employs a dual-stream network to extract features from both occluded and non-occluded samples. Subsequently, the TDC loss alleviates feature homogenization by imposing constraints on the expanded class tokens, encouraging them to learn more diverse and discriminative representations. Finally, the DFF module is proposed to enable the interaction and fusion of features from the two branches.

Figure 1 illustrates the proposed DFLNet architecture, which uses the Vision Transformer (ViT) [30] as its backbone network. The overall architecture primarily consists of the Scene-Level Occlusion (SLO) Strategy, the Token Diversity Constraint (TDC) Loss, and the Diversity Feature Fusion (DFF) Module. During training, the SLO strategy automatically simulates occlusion scenarios based on the relative positions of people and objects in real-world scenes and feeds these into two parallel streams (the occlusion branch and the original branch) for feature extraction. Subsequently, the TDC loss constrains the multi-class tokens expanded by the dual-stream network, thereby enhancing the diversity and distinctiveness of the feature representations. Finally, the DFF module fuses the feature representations from the two branches.

In occluded Re-ID tasks, inference is typically performed by using occluded images as query samples and retrieving identities from a gallery composed of non-occluded images. However, the limited number of occluded samples during training results in the model’s insufficient ability to handle occlusion scenarios. Therefore, simulating occlusion scenarios during training can effectively enhance the model’s generalization ability under occlusion conditions. In the SLO strategy, the original images $\left(X,Y\right)={\left(x_{i},y_{i}\right)}_{i=1}^{B}$ in the dataset are used as input. First, based on the vertical symmetry of the human body, the image is divided horizontally into *N* blocks of equal size. Then, drawing on the spatial semantic relationships between the target person and the occluder in real occlusion scenarios, a simulated occlusion image set $\left(\tilde{X},\tilde{Y}\right)$ is generated to simulate both person-to-person and object-to-human occlusion.

Subsequently, by expanding multiple class tokens, we enable the learning of diverse features for the target pedestrian. The $L_{tdc}$ loss imposes constraints on the multi-class tokens, thereby maximizing the differences between class tokens during the feature learning process. Finally, the DFF module facilitates the interactive fusion of dual-branch diverse feature representations, thereby enhancing the model’s ability to focus on the same target pedestrian.

### 3.2. Scene-Level Occlusion Strategy

Existing occlusion simulation strategies predominantly rely on random pixel replacement or occluder cropping. However, the synthesized samples generated by these approaches fail to adequately capture the diverse occlusion patterns caused by both pedestrians and environmental objects in real-world scenarios. To overcome these limitations, we propose the Scene-Level Occlusion Strategy.

**Figure 2:**
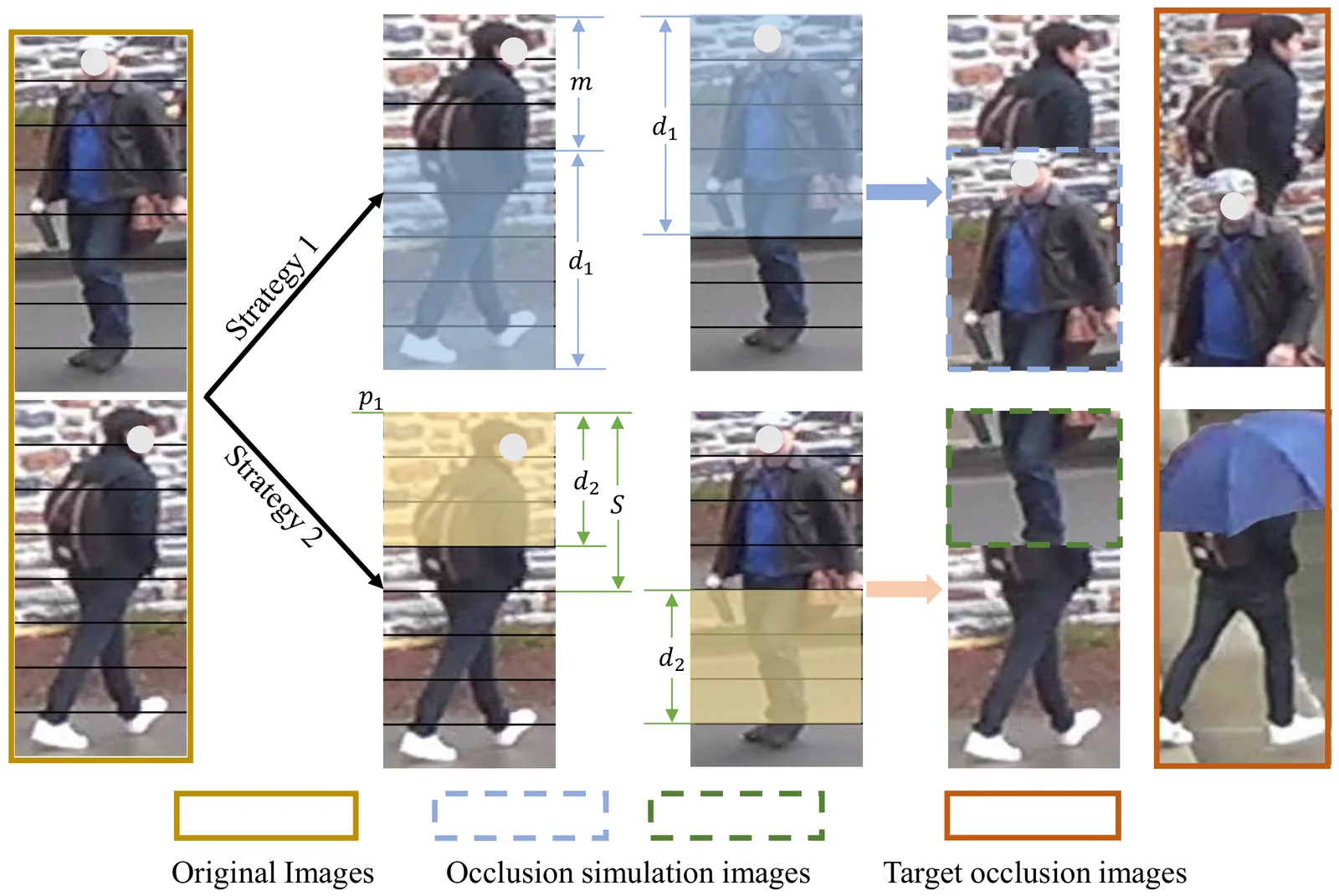
The Specific Implementation Process of the Scene-Level Occlusion Strategy.

**(i) Strategy 1**: Occlusions caused by other pedestrians most often occur in the lower half of the target pedestrian; otherwise, the target pedestrian would be difficult to detect in the field of view. For such occlusion scenarios caused by non-target pedestrians, our approach is to retain the upper half of the target pedestrian to ensure valid visual information, and to use the upper half of the non-target pedestrian as an occlusion region to cover the lower half of the target pedestrian. In the implementation, *m* is defined as the number of upper-body patches retained for the target pedestrian, while $m_{min}$ denotes the minimum number of retained patches required to prevent the target pedestrian from being completely occluded. Given $m_{min}=3$, $m=\mathrm{Randint}(m_{min},N)$, the height of the occlusion region is calculated as $d_{1}=N-m$. Finally, the obtained occlusion region $d_{1}$ is selected to replace the lower part of the target pedestrian, thereby simulating occlusions caused by non-target pedestrians in real-world surveillance scenarios, as illustrated in Figure 2.

**(ii) Strategy 2**: Unlike occlusions caused by non-target pedestrians, which are primarily concentrated in the lower half of the target pedestrian, occlusions caused by obstacles are spatially random, and the occluding objects and the target pedestrian typically belong to different semantic categories. To address this, we adopt an approach that simulates occlusions by introducing semantically distinct body regions. First, a starting patch position $p_{1}=\mathrm{Randint}\left(0,N\right)$ is randomly sampled to determine the initial location of the occlusion region. Then, an occlusion height $d_{2}$ is specified and constrained to satisfy $d_{2}\leq \frac{N}{2}$. To ensure semantic diversity, we set a sliding step $s=\frac{N}{2}$. The actual starting position of the occluder is obtained by shifting $p_{1}$ downward by *s* patches, while the size of the occlusion region remains unchanged as $d_{2}$. Finally, the corresponding region of the target pedestrian image is covered by this occlusion area, as illustrated in Figure 2. It is worth noting that when $p_{1}+s>N$, i.e., the occlusion region exceeds the bottom boundary of the image, a circular wrap-around strategy is adopted to map the exceeding part back to the top region of the image.

During the training phase, for each pedestrian image requiring occlusion simulation, we randomly select either Strategy 1 or Strategy 2 with equal probability (50% each). This balanced sampling mechanism enables the model to adapt simultaneously to both pedestrian and object occlusions, thereby enhancing its robustness in complex scenes.

### 3.3. Token Diversity Constraint Loss

ViT achieves the autonomous flow of information from patch embeddings to class tokens through a hierarchical stacking of self-attention mechanisms. Class tokens act as information collectors in this process. A natural question arises: what if we introduce multiple class tokens, each focusing on different image regions, to capture more diverse feature representations and thereby effectively mitigate the noise interference caused by occlusions. Therefore, we define multiple class tokens $F_{cls}=\left\{f_{cls}^{i}|i=1,2,\ldots ,K\right\}$, where $F_{cls}\in R^{K\times D}$ and *K* denotes the number of class tokens. However, learning features simply by replicating multiple class tokens without any constraints often leads to the class tokens converging to similar feature patterns, causing severe feature homogenization and limiting the model’s ability to distinguish occluded regions. As shown in Figure 1, to address this issue and enhance feature robustness, we propose the Token Diversity Constraint Loss. Unlike a single distance metric, this constraint strategy regularizes class tokens along two dimensions, namely direction and magnitude: it uses cosine distance to maximize semantic differences between tokens and eliminate redundancy, while employing Euclidean distance to constrain the geometric distribution of the feature space and prevent feature divergence. This combined constraint ensures that multi-class tokens can both capture diverse visual cues and maintain stable feature representations.

In the embedding space, we encourage different class tokens to be pushed apart, i.e., maximizing the cosine distance (equivalently minimizing cosine similarity) between their corresponding feature vectors, so that each token attends to distinct semantic regions. The cosine similarity is defined as

(1) $$\begin{array}{c} S_{ij}^{\left(b\right)}=\left|\frac{\langle V_{i}^{\left(b\right)},V_{j}^{\left(b\right)}\rangle }{|V_{i}^{\left(b\right)}{|}_{2}\cdot {\left|V_{j}^{\left(b\right)}\right|}_{2}}\right|,\;\forall i\neq j \end{array}$$

where $V_{i}^{\left(b\right)}$ and $V_{j}^{\left(b\right)}$ are the feature vectors of the *i*-th and *j*-th class tokens in the *b*-th sample, respectively. 〈·,·〉 denote the inner product, and ${\left\|\cdot \right\|}_{2}$ denotes the L2 norm. To reduce computational redundancy and avoid autocorrelation interference, we retain only the upper triangular part of the similarity matrix (i.e., *j* > *i*), forming the following set:

(2) $$T=\left\{S_{ij}^{\left(b\right)}|1\leq i<j\leq K\right\}$$

where $|T|=\frac{K(K-1)}{2}$. The traditional approach involves averaging all token pairs in set T, but this tends to cause the model to prioritize optimizing simple sample pairs that already exhibit distinct differences, while neglecting difficult sample pairs with high similarity. Inspired by the principle of entropy maximization, we introduce a Softmax-based adaptive weight allocation mechanism that allows the model to automatically focus on difficult sample pairs. The specific calculation formula is as follows:

(3) $$w_{ij}^{\left(b\right)}=\frac{\exp\left(S_{ij}^{\left(b\right)}\right)}{\sum_{(m,n)\in T}\exp\left(S_{mn}^{\left(b\right)}\right)}$$

Therefore, the cosine constraint loss is defined as

(4) $$L_{cos}=\frac{1}{2B|T|}\sum_{b=1}^{2B}\sum_{(i,j)\in T}w_{ij}^{\left(b\right)}\cdot S_{ij}^{\left(b\right)}$$

Cosine distance primarily measures the directional differences between class tokens, but it does not account for differences in the magnitude of feature vectors. In the occlusion-aware pedestrian re-identification task, different tokens may point in similar directions yet exhibit significant differences in feature magnitude; relying solely on cosine distance constraints makes it difficult to fully capture the complementary features between direction and magnitude. Therefore, we introduce an Euclidean distance term as an auxiliary regularization constraint. Unlike the diversity constraint, which aims to maximize differences, this constraint is designed to maintain the compactness and stability of the feature space. By minimizing the Euclidean distance between class tokens, we force the model to learn discriminative directional features while limiting the excessive spread of feature vectors in geometric space, thereby preventing abnormal fluctuations in feature magnitude and enhancing the robustness of the representations. Specifically, the Euclidean distance $E_{ij}^{\left(b\right)}$ is calculated as follows:

(5) $$E_{ij}^{\left(b\right)}={\left|v_{i}^{\left(b\right)}-v_{j}^{\left(b\right)}\right|}_{2}$$

To avoid double counting and maintain semantic independence, we select elements from the lower triangular part of the matrix to construct a sparse distance set, i.e., all $E_{ij}^{\left(b\right)}$ satisfying i>j. The corresponding sparse distance set is defined as

(6) $$\varepsilon =\left\{E_{ij}^{\left(b\right)}|1\leq j<i\leq K\right\}$$

To enforce the model to simultaneously align these hard samples in two distinct metric subspaces, preventing the model from separating samples in one space while remaining confused in the other, we adopt the same weighting strategy $u_{ij}^{\left(b\right)}$ as the cosine distance. This enables the loss function to adaptively focus on token pairs that are already close in the feature space, thereby imposing a more refined compactness constraint. The Euclidean distance loss is formulated as

(7) $$L_{eu}=\frac{1}{2B|\varepsilon |}\sum_{b=1}^{2B}\sum_{(i,j)\in \varepsilon }u_{ij}^{\left(b\right)}\cdot E_{ij}^{\left(b\right)}$$

In summary, the Token Diversity Constraint Loss is composed of both the cosine distance, which focuses on geometric aspects, and the Euclidean distance, which focuses on spatial compactness:

(8) $$L_{tdc}=L_{cos}+\alpha L_{eu}$$

where α is a hyperparameter for balancing $L_{eu}$. This combined strategy ensures that the class tokens not only exhibit high semantic discriminability in the directional space but also maintain favorable numerical stability in the feature space.

### 3.4. Diversity Feature Fusion Module

Although single-branch class tokens can learn differentiated feature representations under diversity constraints, their representational capacity remains limited by the branch’s own receptive field and attention distribution. Specifically, because different branches share the same network architecture but receive different image inputs, they often focus on different discriminative regions within an image. However, existing methods typically fuse multi-branch features using simple weighted sums or direct averaging, an approach that has two limitations: First, simple feature-level fusion struggles to balance the discriminative information from the two branches, which can easily lead to noise contamination caused by occluded global features. Second, since different branches contribute to the final classification decision to varying degrees, treating all branches equally weakens the dominant role of strongly discriminative branches.

These are then fed separately into the Transformer Encoder to establish global correlations among class tokens. This decoupled design allows each branch to fully exploit contextual information within its specific semantic space: the original branch focuses on extracting complete, structured features, while the occlusion branch is dedicated to uncovering locally robust features. The two branches operate in parallel without interfering with one another, enabling class tokens to adaptively aggregate discriminative information from different image patches.

To fully leverage the complementary strengths of the two branches while avoiding noise interference, we propose the Diversity Feature Fusion Module, as shown in Figure 1. Unlike traditional cross attention mechanisms that enforce feature blending, we adopt a strategy that combines parallel enhancement with decision-level complementarity. The input to this module consists of multi-class tokens from the dual branches that have been optimized via $L_{tdc}$. These are then fed separately into the Transformer Encoder to establish global correlations among class tokens. This decoupled design allows each branch to fully exploit contextual information within its specific semantic space: the original branch focuses on extracting complete, structured features, while the occlusion branch is dedicated to uncovering locally robust features. The two branches operate in parallel without interfering with one another, enabling class tokens to adaptively aggregate discriminative information from different image patches. The specific formula is as follows:

(9) $$F_{O}=\mathrm{Normalize}\left(\mathrm{MLP}\left(\mathrm{Softmax}\left(\frac{F_{cls}^{O}⊗{F_{cls}^{O}}^{T}}{\sqrt{D}}\right)⊗F_{cls}^{O}\right)\right)$$

(10) $$F_{H}=\mathrm{Normalize}\left(\mathrm{MLP}\left(\mathrm{Softmax}\left(\frac{F_{cls}^{H}⊗{F_{cls}^{H}}^{T}}{\sqrt{D}}\right)⊗F_{cls}^{H}\right)\right)$$

To fully preserve the discriminative information from the two branches and enable the subsequent utilization of the complementary features from both perspectives, we concatenate $F_{O}$ and $F_{H}$ as shown in the following formula:

(11) $$T=\left\{Concat\left(F_{O},F_{H}\right)\right\}$$

This concatenation operation is not merely a simple stacking of information but rather a highly efficient mechanism for feature complementarity. Compared to direct weighted averaging (which tends to smooth out feature differences) or early cross-attention methods (which tend to introduce occlusion noise), our approach achieves more robust feature complementarity while preserving feature purity. Subsequently, to further extract the most salient semantic information from the features, a pooling operation is applied to obtain $\tilde{T}$. Given that the ArcFace loss introduces an additive angular margin penalty in the hyperspherical embedding space, it not only enlarges inter-class distances but also significantly reduces intra-class variance. The features extracted from the occlusion branch and the global branch often suffer from inconsistent magnitudes and distribution shifts. To address this issue, we employ the ArcFace loss to normalize the features and impose angular constraints, effectively eliminating the interference caused by magnitude discrepancies. This forces the occluded and non-occluded features of the same identity to be aligned in the angular space, thereby enhancing identity discriminability while alleviating the feature distribution gap between the two branches. The formula is as follows:

(12) $$L_{arc}=-\frac{1}{2B}\sum_{i=1}^{2B}\log\frac{e^{\varepsilon (\cos\left(\theta_{y_{i}}+m\right))}}{e^{\varepsilon (\cos\left(\theta_{y_{i}}+m\right))}+\sum_{\begin{array}{c} j=1\\ j\neq y_{i} \end{array}}^{N}e^{\varepsilon (\cos\left(\theta_{j}\right))}}$$

where *B* denotes the number of identities in the training batch; *N* represents the total number of classes in the dataset; $y_{i}$ indicates the ground-truth class label of the (i)-th sample; $\theta_{y_{i}}$ denotes the angle between the feature vector of the *i*-th sample and its corresponding class center; ε is the scaling factor used to adjust the dynamic range of the cosine values; and *m* is the additive angular margin penalty used to increase the inter-class distance.

### 3.5. Objective Function

During the training stage, our method is optimized using the identity ID loss and triplet loss, formulated as follows:

(13) $$L_{ID}=-\frac{1}{2B}\sum_{i=1}^{2B}\log\left(p(y_{i}|x_{i})\right)$$

(14) $$L_{tri}=\log\left[1+\exp\left(\|f_{a}-f_{p}{\|}_{2}^{2}-{\left\|f_{a}-f_{n}\right\|}_{2}^{2}\right)\right]$$

where 2B denotes the number of images in the dual-output setting.

Eventually, the overall objective function can be expressed as follows:

(15) $$L_{total}=L_{ID}+L_{tri}+L_{tdc}+\beta L_{arc}$$

where β is the hyper-parameter for balancing $L_{arc}$.

## 4. Experiment

### 4.1. Datasets and Evaluation Metrics

We evaluated our model on two occluded datasets, two partial datasets, and two holistic datasets.

**Occluded-DukeMTMC** [15]: This dataset is constructed based on DukeMTMC-ReID. The training set contains 15,618 images of 201 pedestrian identities. The query set consists of 2210 occluded images from 519 identities, while the gallery set includes 17,661 images of 1110 identities.

**Occluded-ReID** [31]: This dataset is captured using mobile cameras from multiple viewpoints and contains 2000 images of 200 pedestrian identities. Each identity consists of 5 holistic images and 5 occluded images.

**Partial-REID** [32]: This dataset is collected from six different cameras on a university campus and contains 600 images of 60 pedestrian identities. Each identity consists of 5 holistic images and 5 partial images, featuring diverse backgrounds, viewing angles, and occlusion types.

**Partial-iLIDS** [33]: This dataset was collected in an airport environment and contains a total of 238 images representing 119 pedestrian identities. For each identity, there is one query image and one library image.

**Market-1501** [34]: It is a widely used benchmark dataset for holistic person re-identification, collected from six different surveillance cameras. The training set contains 12,936 images of 751 pedestrian identities. The query set includes 3368 images from 750 identities, while the gallery set contains 19,732 images of the same 750 identities.

**DukeMTMC-ReID** [35]: The training set contains 16,522 images across 702 identities, the query set contains 2228 images, and the gallery set contains 17,661 images across 1110 identities.

**Evaluation Metrics**: Following the standard evaluation protocol for person Re-ID, we adopt the Cumulative Matching Characteristic (CMC) and mean Average Precision (mAP) as evaluation metrics. The values of the CMC curve at Rank-1, Rank-3, Rank-5, and Rank-10 are denoted as R-1, R-3, R-5, and R-10, respectively.

### 4.2. Implementation Details

We adopt the ViT(ViT-B/16) backbone pre-trained on ImageNet. All input images are resized to 256×128. To preserve finer-grained spatial information, we set the stride of the patch embedding to 11×11. During training, random cropping, horizontal flipping, random erasing, and random grayscale conversion are applied as basic data augmentation techniques. We use SGD as the optimizer with a momentum of 0.9. The initial learning rate is set to 0.008 and is decayed using a cosine annealing schedule with a minimum learning rate of $1\times 10^{-4}$. The batch size is set to 64, containing 16 identities per batch with 4 images sampled per identity. The number of class tokens *K* is set to 5. For ArcFace loss, the scaling factor ε and margin *m* are set to 30 and 0.5, respectively. The model is trained for 120 epochs. The DFF module extracts semantic features from the *L*-th layer of the Transformer Encoder, with the feature dimension maintained at 768. The weighting coefficients α and β in the overall objective function are both set to 0.1. All experiments are conducted on NVIDIA A40 GPU.

### 4.3. Comparison with the State-of-the-Art Methods

**Results on Occluded Datasets**: To validate the effectiveness of the proposed DFLNet method, we conducted extensive experiments on the Occluded-DukeMTMC and Occluded-ReID datasets, comparing our method with current mainstream approaches (namely, CNN-based, auxiliary model-based, and Transformer-based methods). Among the CNN-based methods, QPM [14] and PRE-Net [5] primarily focus on optimizing the perceptual capacity of local features to enhance feature discrimination in visible regions. Among the auxiliary model-based methods, PGFL-KD [36] employs a knowledge distillation strategy to guide the learning of body part features, while PFD [16] utilizes a Transformer to decouple and match human body features; both methods attempt to compensate for information loss caused by occlusion through pose estimation. Among the Transformer-based methods, OAT [20] focuses on capturing higher-order correlations between human body regions, whereas MAHATMA [21] extracts finer visual features by aggregating hierarchical image patches.

Experimental results show that Transformer-based methods offer significant advantages in improving model performance, and our method inherits this characteristic. As shown in Table 1, our proposed DFLNet demonstrates highly competitive performance on two occluded pedestrian re-identification datasets. In particular, on the Occluded-DukeMTMC dataset, the DFLNet model achieved a Rank-1 accuracy of 76.0% and an mAP of 63.9%, outperforming the vast majority of existing occluded re-ID methods. It is worth noting that our proposed method is able to achieve excellent performance in complex occlusion scenarios without relying on additional pre-trained models.

**Results on Holistic Datasets**: Although DFLNet is originally designed to address occluded person re-identification, we further evaluate its generalization capability on two holistic benchmarks by comparing it with existing methods, including both CNN-based and Transformer-based approaches. As shown in Table 2, DFLNet achieves state-of-the-art performance on Market-1501, with 95.9% Rank-1 accuracy and 90.2% mAP. It also demonstrates strong performance on the DukeMTMC-ReID dataset. The consistently superior results on these non-occlusion-dominated holistic benchmarks further validate that DFLNet maintains strong robustness and generalization ability across diverse visual scenarios.

**Results on Partial Datasets**: To further validate DFLNet’s generalization ability when processing incomplete pedestrian images, we conducted experiments on the Partial-REID and Partial-iLIDS datasets. Since these two datasets do not provide dedicated training samples, we followed existing work and used the Market-1501 dataset as the source domain for model training. As shown in Table 3, the proposed method achieved highly competitive performance on both partial datasets, with Rank-1 and Rank-3 metrics reaching 82.3%/89.0% and 79.0%/87.4%, respectively. These results clearly demonstrate that even in the absence of training data specific to a given scenario, the method can maintain strong feature extraction capabilities through cross-dataset learning.

### 4.4. Ablation Studies

In this section, we conduct systematic ablation studies on the Occluded-DukeMTMC dataset to evaluate the effectiveness of each component. Specifically, we analyze the Scene-Level Occlusion (SLO) Strategy, the Token Diversity Constraint (TDC) Loss, and the Diversity Feature Fusion (DFF) Module.

**Effectiveness of the SLO Strategy**: By comparing the Baseline with “w/SLO” in Table 4, the performance gains brought by the Scene-Level Occlusion Strategy (SLO) can be clearly observed. Specifically, Rank-1 accuracy and mAP are significantly improved from 64.4% and 57.3% to 73.0% and 61.9%, respectively. These results demonstrate that the proposed strategy effectively simulates complex and diverse occlusion scenarios encountered in real-world environments during training, forcing the model to learn more discriminative feature representations, thereby substantially enhancing its robustness and generalization capability in complex occlusion scenarios.

**Figure 3:**
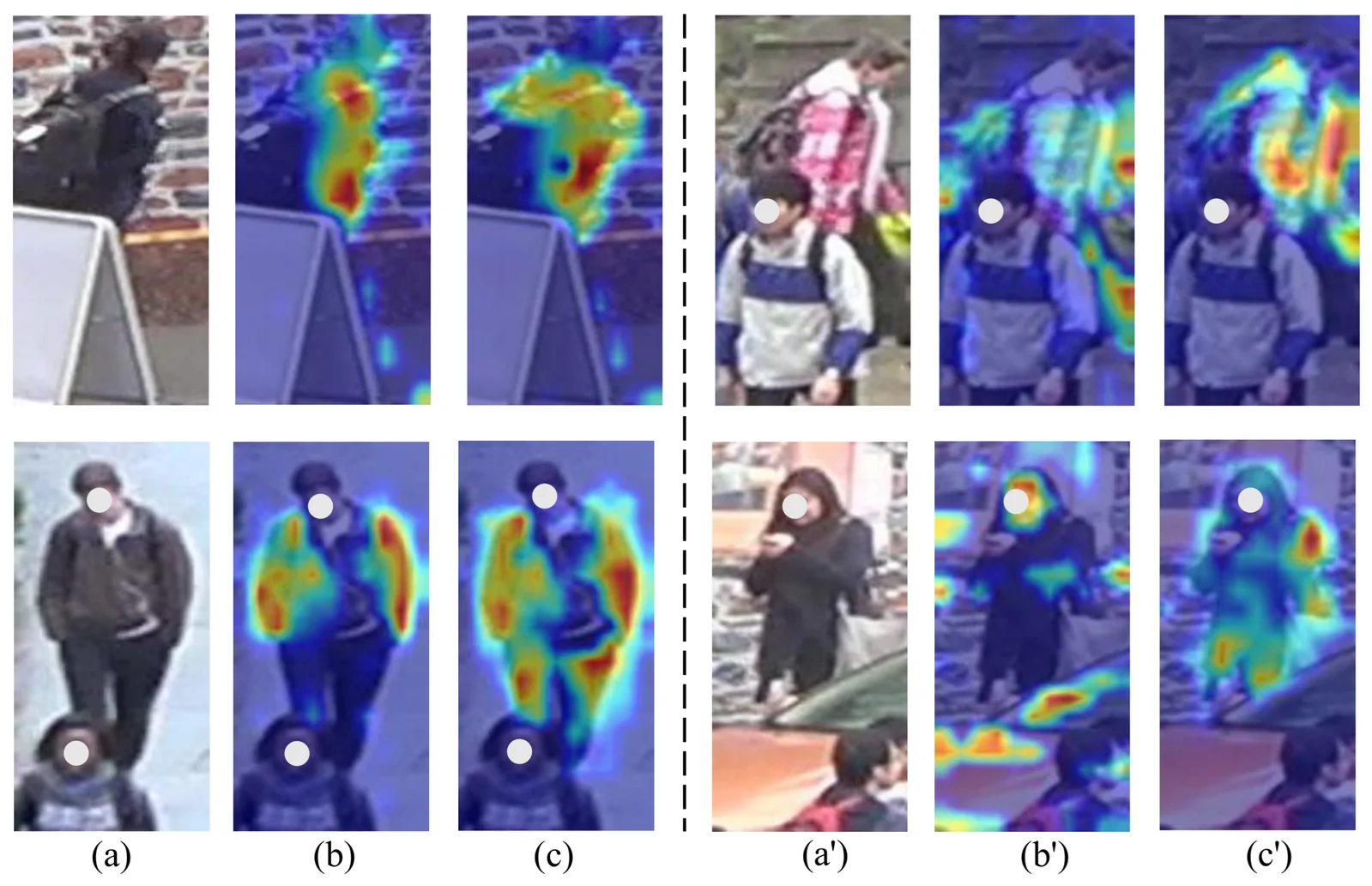
Grad-CAM visualization of attention maps on Occluded-DukeMTMC. (**a**,**a’**) Input image. (**b**,**b’**) Baseline. (**c**,**c’**) Baseline + SLO.

Figure 3 visually illustrates the differences in attention distribution between the baseline model and the model with the SLO strategy using Grad-CAM heatmaps. It can be observed that, under various occlusion scenarios, the baseline model is often prone to interference from occluding objects, resulting in a more diffuse focus; in contrast, after introducing the SLO strategy, the model is able to focus more precisely on the effective semantic features of pedestrians. This not only achieves effective separation between obstacles and target pedestrians but also successfully guides the network to concentrate its attention on visible body parts, thereby significantly improving the accuracy of feature extraction.

**Figure 4:**
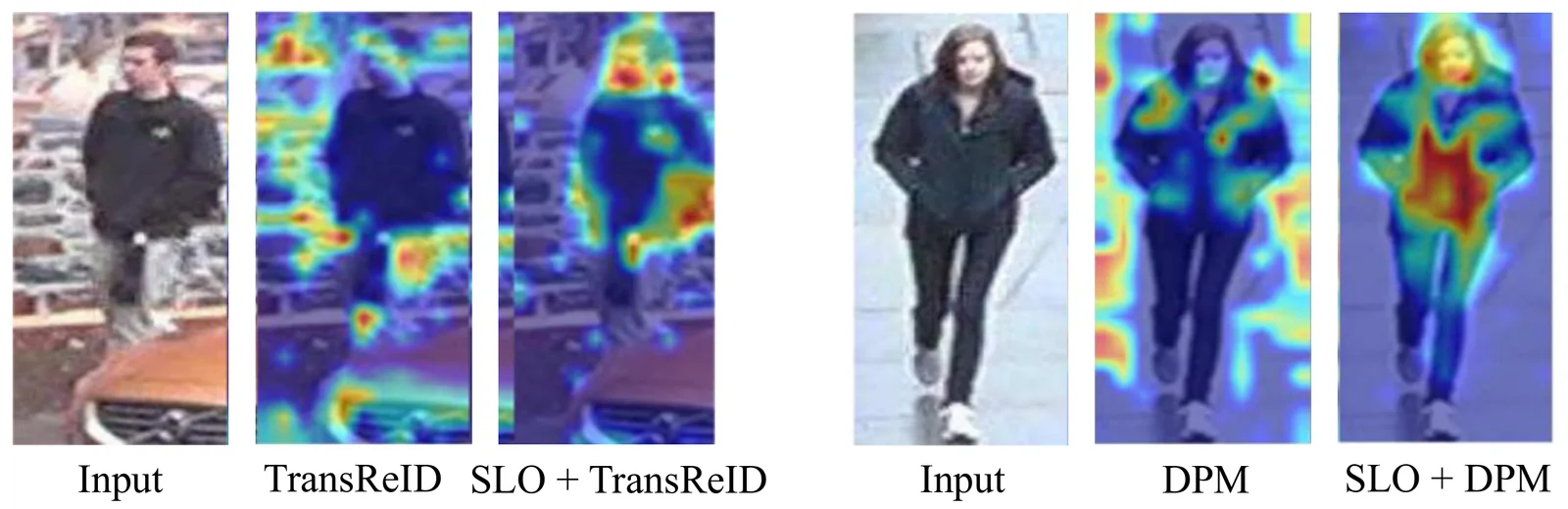
Grad-CAM visualization of attention maps on Occluded-DukeMTMC.

Table 5 compares different occlusion simulation methods, all of which use ViT as the backbone network. Among these, NFDM [25] focuses on removing occlusions rather than learning feature representations under occlusion. In real-world scenarios, occlusions are an objective reality, and models must be capable of extracting effective features even in the presence of occlusions. CutMix [52] is an unsupervised pixel-level blending method that often disrupts the semantic structure of pedestrians (for example, by forcibly overlaying background textures onto a pedestrian’s legs), causing the model to learn incorrect associations. OIA [27] is limited by a small dataset and lacks the ability to generalize to complex real-world environments; furthermore, it often involves simple pixel-level superimposition, lacking deep semantic interactions. PKOS [47] primarily relies on an instance-based copy-and-paste strategy, which often ignores the spatial relationship between the target pedestrian and the occluding object, making it prone to introducing semantic noise and interfering with feature extraction. In contrast, the occlusion enhancement strategy we propose deeply integrates semantic priors and spatial knowledge from real-world scenes, enabling comprehensive simulation of the diverse occlusions caused by pedestrians and objects. Experimental results show that the proposed SLO strategy significantly outperforms all the above comparison methods, fully validating its effectiveness in occlusion simulation. To verify the generality and scalability of the proposed SLO strategy, we integrated it into various existing mainstream methods (while fully preserving all core contributions of these original methods). As shown in the lower half of Table 5, the performance of each model was further improved after introducing the SLO strategy. In addition, we used Grad-CAM heatmaps to visually analyze the attention distributions before and after the insertion of the SLO strategy, as shown in Figure 4. This result strongly confirms that the SLO strategy possesses excellent generalization capabilities and can be seamlessly integrated with and enhance various pedestrian re-identification models.

**Effectiveness of the TDC Module**: As shown in Table 4, the transition from “Baseline” to “w/TDC” demonstrates that introducing the TDC loss improves Rank-1 and mAP by 4.0% and 3.6%, respectively. Furthermore, when TDC is integrated on top of the SLO strategy, the performance is further boosted from 73.0% and 61.9% to 74.9% and 63.1%, respectively. These results further validate the effectiveness of TDC in enhancing feature discriminability, as well as its strong compatibility with the SLO strategy.

**Figure 5:**
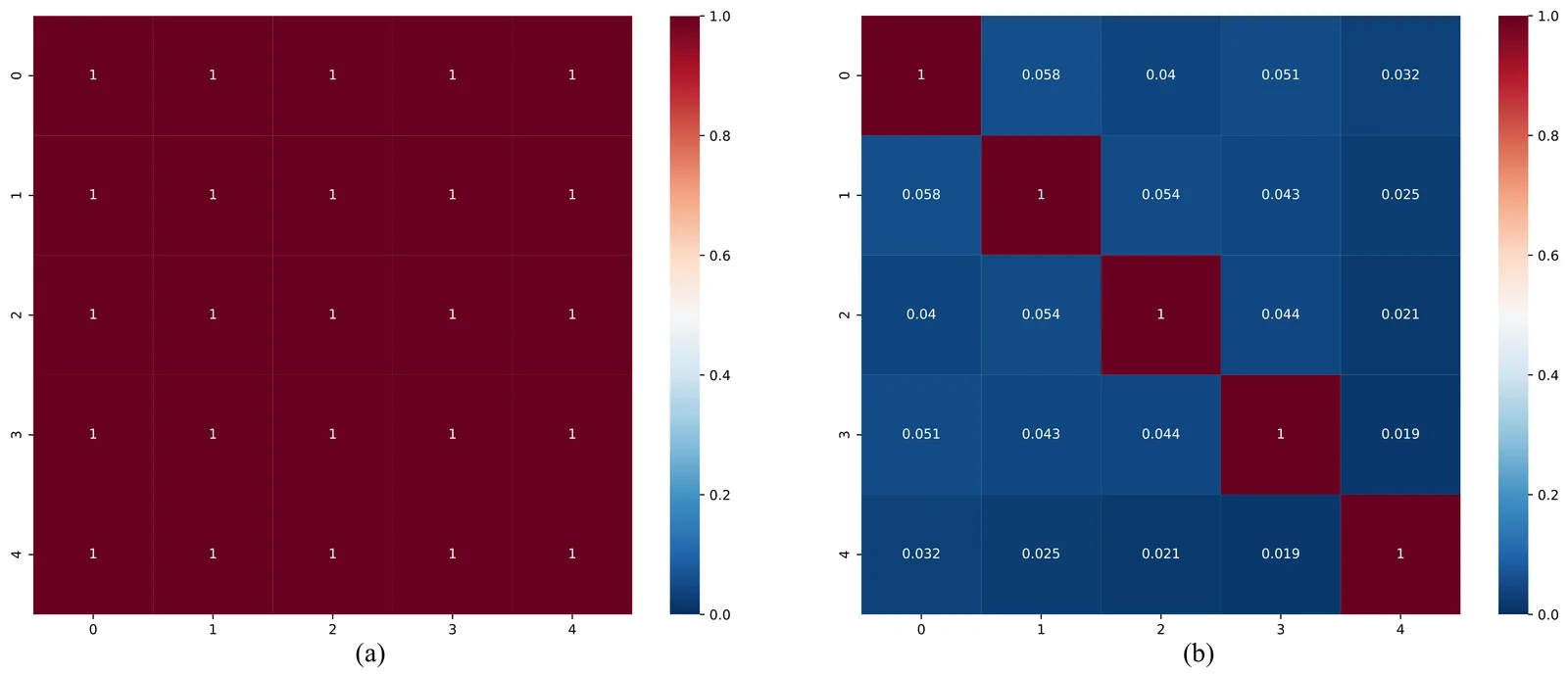
Feature correlations between multiple class tokens. (**a**,**b**) Training without/with TDC loss.

As shown in Table 6, we conduct an ablation study on the TDC module using the Occluded-DukeMTMC dataset. The results indicate that employing multiple class tokens (MCT) consistently improves the model’s performance. This improvement can be attributed to the ability of multiple class tokens to learn more diverse feature representations. Simply increasing the number of class tokens can theoretically expand the feature capturing capacity; however, without proper constraints, naive duplication often leads to redundancy in the feature space. To address this issue, we introduce the $L_{tdc}$ to explicitly enforce the learning of discriminative and diverse representations. As shown in Table 6, applying both cosine distance and Euclidean distance to the multi-class tokens further improves model performance, and the model achieves optimal performance when both are applied simultaneously. This combined strategy ensures that the class tokens are not only highly distinguishable semantically but also maintain good numerical stability in the feature space. Figure 5 visually illustrates the effect of this constraint using feature correlation matrices. Without applying $L_{tdc}$ (Figure 5a), the class tokens exhibit high similarity and strong redundancy. In contrast, after introducing the constraint (Figure 5b), the correlation between features is significantly reduced, indicating that $L_{tdc}$ guides different tokens to focus on distinct feature representations of pedestrians.

**Effectiveness of the DFF Module**: The results in Table 4 further confirm the effectiveness of the DFF module in improving model performance. Specifically, incorporating this module leads to gains of 1.1% in Rank-1 accuracy and 0.8% in mAP. These improvements demonstrate that DFF effectively facilitates interactive learning between the original and occluded branches, enabling the model to more accurately match the same target identity across different scenarios and achieve better complementarity and enhancement of global and local information. We compared the proposed DFF module with traditional fusion strategies, including Direct Concatenation and Weighted Average. As shown in Table 7, our method achieved the best performance. The poor performance of the weighted average indicates that a simple linear combination cannot handle the significant distribution differences between non-occluded and occluded features. At the same time, the performance improvement achieved compared to direct concatenation validates the necessity of the independent Transformer encoding stage, which effectively enhances the robustness of the features before the final embedding integration.

**Figure 6:**
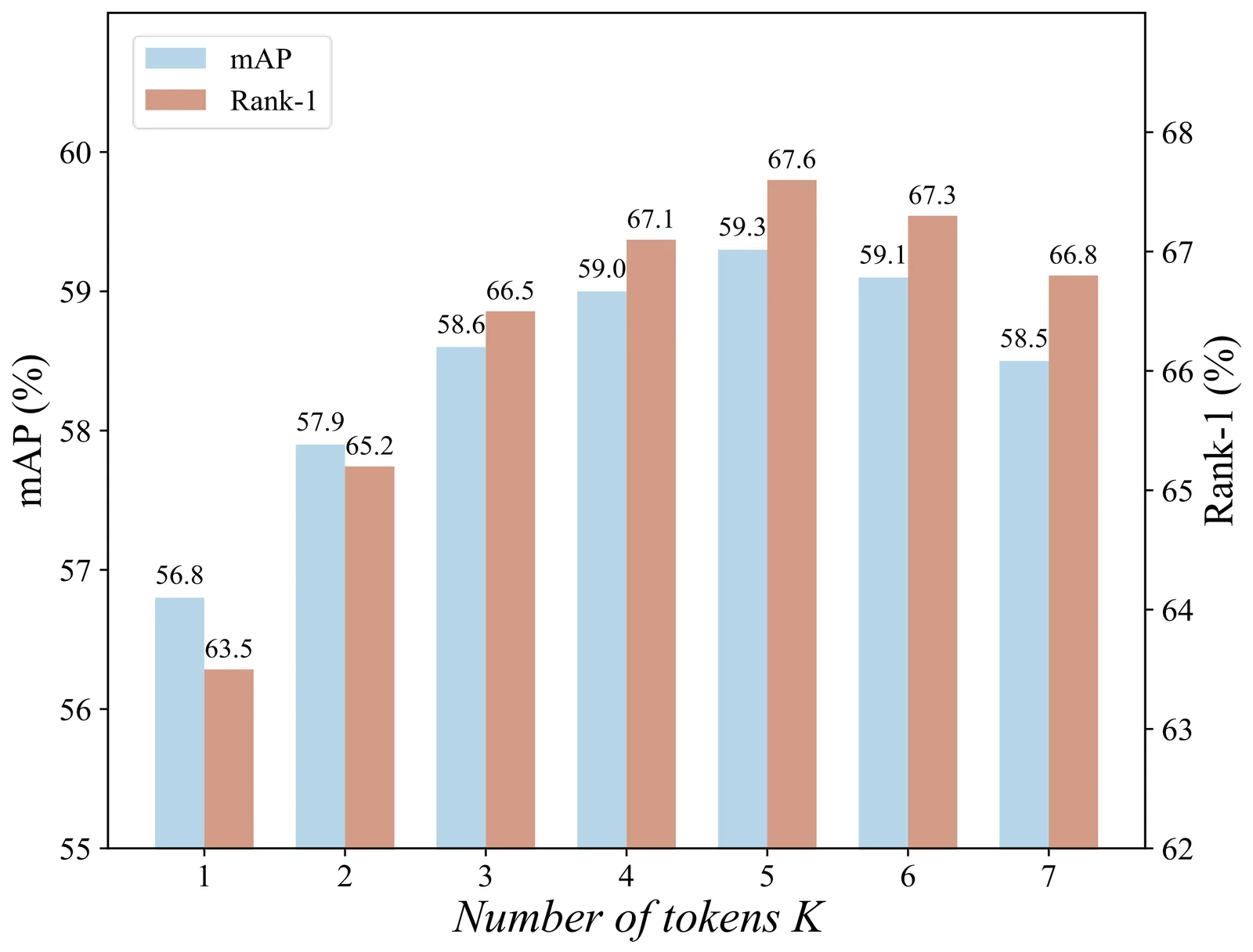
Ablation analysis of the number of class tokens on Occluded-DukeMTMC.

**Influence of the Number of Class Tokens**: Figure 6 illustrates the impact of the number of class tokens on model performance on the Occluded-DukeMTMC dataset. The experimental results show that when K=1, the model achieves the worst performance due to the limited expressive capacity of a single token. As *K* increases, both Rank-1 accuracy and mAP consistently improve, indicating that introducing multiple class tokens enables the model to capture richer semantic information of pedestrians. However, when *K* exceeds a certain threshold, the performance begins to degrade. This is because excessive class tokens introduce redundant features and increase the difficulty of discriminating highly similar token representations, leading to unstable optimization. When K=5, the model achieves the best overall performance, with Rank-1 accuracy reaching 67.6% and mAP reaching 59.3%. Therefore, the number of class tokens is set to K=5 in all final experiments.

**Figure 7:**
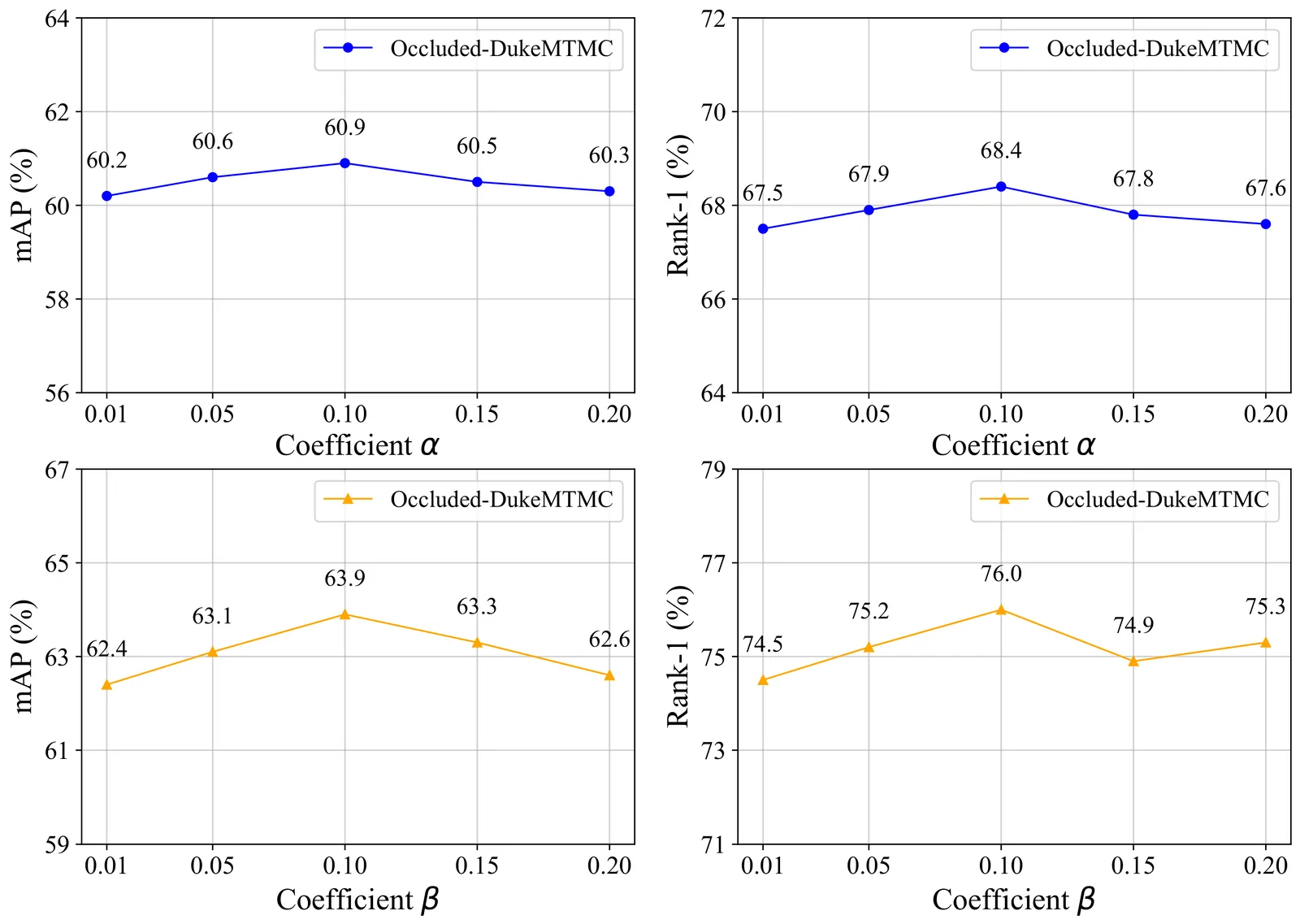
Ablation analysis of hyper-parameters α and β on Occluded-DukeMTMC.

**Influence of hyper-parameters α and β**: To balance the influence of different loss functions during training, we introduced the hyper-parameters α and β. Based on the setting K=5, we conduct detailed ablation studies on the Occluded-DukeMTMC dataset to determine their optimal configurations. We first analyze α, which controls the strength of the Euclidean distance constraint. As shown in the upper part of Figure 7, the model achieves its best performance when α=0.1. This is because cosine similarity primarily measures directional differences between feature vectors but cannot effectively constrain feature magnitudes. Introducing the Euclidean distance constraint compensates for this limitation by further separating class tokens in terms of feature scale. When α is too small, the Euclidean constraint is insufficient to eliminate feature redundancy. Conversely, overly large values of α excessively enforce spatial separation, thereby disrupting the inherent intra-class compactness. Next, with α fixed, we investigate the effect of β, which is used to balance the angular distance between occluded and non-occluded features. The results show that when β is small, the angular separation between features is insufficient. In contrast, excessively large β forces an overly wide angular margin, causing features to cross decision boundaries and degrading recognition accuracy. As illustrated in the lower part of Figure 7, the best performance is achieved when β=0.1.

### 4.5. Visualization

**Figure 8:**
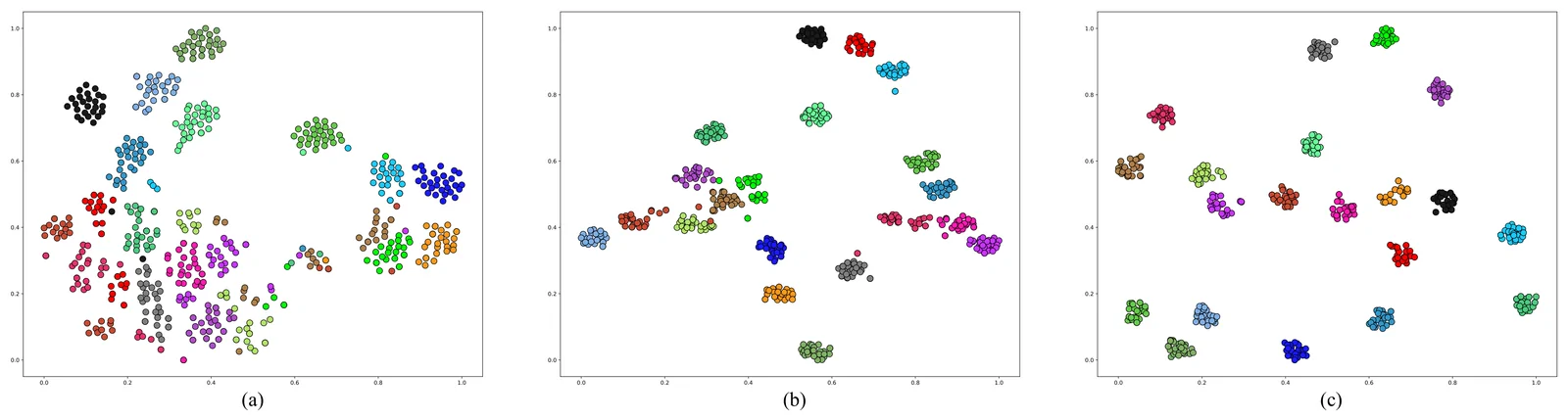
Visual analysis of feature distribution on the Occluded-DukeMTMC dataset. (**a**) Baseline distribution. (**b**) TDC distribution. (**c**) Ours distribution.

**Figure 9:**
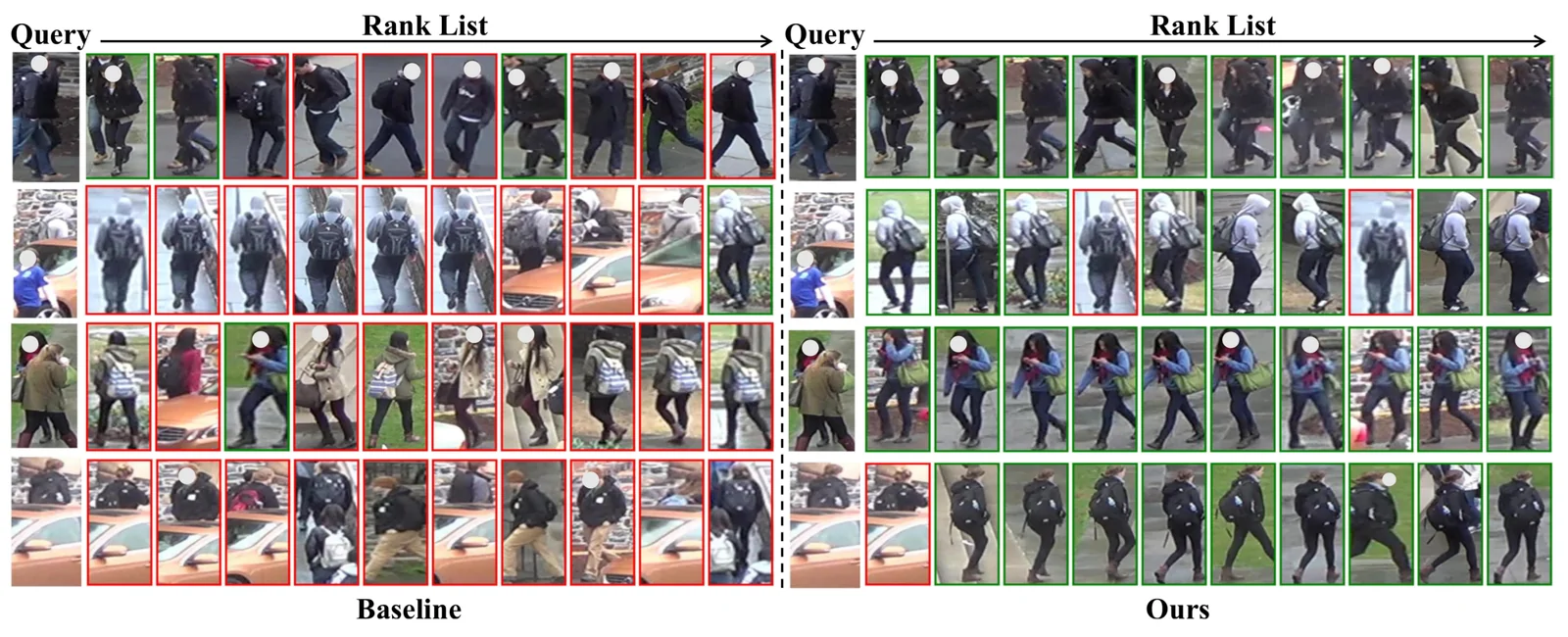
Visualization comparison of our DFLNet method and baseline rank-10 retrieval result on Occluded-DukeMTMC.

To visually validate the effectiveness of the method proposed in this paper for feature learning, we used t-SNE to map high-dimensional features onto a two-dimensional plane for visualization on the Occluded-DukeMTMC dataset. The results are shown in Figure 8. Each colored cluster in the figure represents a specific pedestrian identity. As can be seen in Figure 8a, the feature distribution generated by the baseline model is relatively sparse, and there is significant overlap between different identities (particularly in the lower-left corner and the central region), indicating that the baseline features lack sufficient discriminative power in complex scenes. In contrast, Figure 8b shows the feature distribution after introducing the TDC loss. As can be seen, the cluster structures for each category have become more compact, and the overlap between classes has been significantly reduced, demonstrating the critical role of this loss function in constraining the feature space and enhancing feature separability. Finally, Figure 8c displays the feature distribution of the proposed complete model. In this space, features representing different identities form distinct, highly cohesive, and independent clusters, with the inter-class separation significantly increased. This result strongly demonstrates that the overall framework proposed in this paper is capable of learning feature representations that are more robust and discriminative. To further compare our method with baseline approaches, we selected images with different types of occlusions (such as non-target pedestrian occlusions and obstacle occlusions) from the Occluded-DukeMTMC dataset to conduct a visual comparison of Rank-10 retrieval results, intuitively demonstrating the significant advantages of our proposed method over the baselines. In the Figure 9, green boxes represent correct matches, while red boxes represent incorrect matches. As shown in the figure, under various complex occlusion conditions, the baseline method often struggles to effectively distinguish the target pedestrian from the occluding objects, leading to confusion between the target region and background noise during feature extraction. In contrast, the proposed method is able to more sensitively capture and focus on the visible regions of the target pedestrian, effectively suppressing feature interference caused by occlusion. This comprehensive focus on key visible information enables the model to accurately retrieve the target pedestrian even under severe occlusion conditions.

### 4.6. Complexity Analysis

To provide a more comprehensive evaluation of the proposed DFLNet, we compare it with the baseline in terms of both model complexity and performance. Model complexity is evaluated using the number of parameters, FLOPs, and FPS. As shown in Table 8, the proposed DFF module reuses the weights of the internal Transformer layers in the ViT backbone without introducing additional convolutional kernels or fully connected layers. Consequently, the number of model parameters remains unchanged. In terms of inference efficiency, although the FLOPs increase slightly (from 22.530G to 23.481G, an increase of approximately 4%), resulting in a marginal decrease in FPS from 287.9 to 275.1; this overhead is well within an acceptable range. More importantly, this negligible computational cost yields substantial performance gains, improving Rank-1 accuracy by 11.6% and mAP by 6.6%. These results further demonstrate the effectiveness of the proposed method.

## 5. Conclusions

In this paper, we propose a Diversity Feature Learning Network (DFLNet) for occluded person re-identification. First, we design a Scene-Level Occlusion (SLO) Strategy that performs data augmentation by simulating the relative spatial relationships between target pedestrians and occluders in real-world scenarios, thereby effectively improving the model’s adaptability to diverse occlusion environments. Second, we introduce a Token Diversity Constraint (TDC) loss, which imposes constraints on the expanded class tokens to alleviate feature homogenization and encourage the learning of more discriminative and diverse feature representations. Finally, we propose a Diversity Feature Fusion (DFF) module to facilitate the interaction and fusion of dual-branch features, enabling the model to accurately focus on consistent identity information under both occluded and non-occluded conditions. Extensive experimental results demonstrate the effectiveness of the proposed framework.

## Figures and Tables

**Table 1:** Quantitative comparison with state-of-the-art methods on Occluded-DukeMTMC (%) and Occluded-ReID (%). Bold indicates the best results, while underlining indicates sub-optimal results.

Methods	Reference	Occluded-DukeMTMC		Occluded-ReID	
		Rank-1	mAP	Rank-1	mAP
PCB [13]	ECCV18	42.6	33.7	41.3	38.9
DSR [33]	CVPR18	40.8	30.4	72.8	62.8
OAMN [7]	ICCV21	62.6	46.1	-	-
QPM [14]	TMM22	64.4	49.7	-	-
PRE-Net [5]	TCSVT23	68.3	55.2	-	-
RTGAT [6]	TIP23	61.0	50.1	71.8	51
HOReID [9]	CVPR20	55.1	43.8	80.3	70.2
PGFL-KD [36]	ACMMM21	63.0	54.1	80.7	70.3
PFD [16]	AAAI22	69.5	61.8	81.5	82.0
RFCNet [37]	TPAMI22	63.9	54.5	-	-
FRT [38]	TIP22	70.7	61.3	80.4	71.0
MSDPA [39]	ACMMM22	70.4	61.7	81.9	77.5
BPBReID [10]	WACV23	66.7	54.1	76.9	68.6
HCGA [40]	TIP23	70.2	57.5	-	-
TransReID [18]	ICCV21	66.4	59.2	-	-
DPM [19]	ACMMM22	71.4	61.8	85.5	79.7
RGANet [41]	TIFS23	71.6	62.4	86.4	80.0
DRL-Net [26]	TMM23	65.8	53.9	-	-
SAP [42]	AAAI23	70.0	62.2	83.0	76.8
DPM-SPT [43]	AAAI24	74.7	63.0	**87.8**	81.8
OAT [20]	TIP24	71.8	62.2	82.6	78.2
FCFormer [27]	TMM24	73.0	63.1	83.6	**85.7**
MAHATMA [21]	TCSVT25	73.3	62.3	85.8	79.5
UD-Gaussian [44]	TIP26	71.2	62.0	85.8	80.1
**DFLNet (Ours)**		**76.0**	**63.9**	86.5	82.1

**Table 2:** Quantitative comparison with state-of-the-art methods on Market-1501 (%) and DukMTMC-ReID (%). Bold indicates the best results, while underlining indicates sub-optimal results.

Methods	Reference	Market-1501		DukeMTMC-ReID	
		Rank-1	mAP	Rank-1	mAP
PGFA [15]	ICCV19	91.2	76.8	82.6	65.5
HOReID [9]	CVPR20	94.2	84.9	86.9	75.6
SORN [45]	TCSVT20	94.8	84.5	86.9	74.1
OAMN [7]	ICCV21	93.2	79.8	86.3	72.6
HCGA [40]	TIP22	95.2	88.4	-	-
FED [25]	CVPR22	95.0	86.3	89.4	78.0
CAAO [29]	TIP23	95.3	88.0	89.8	80.9
RTGAT [6]	TIP23	95.3	88.2	89.1	80.2
TransReID [18]	ICCV21	95.2	88.9	90.7	82.0
DPM [19]	ACMMM22	95.5	89.7	91.0	82.6
DRL-Net [26]	TMM22	94.7	86.9	88.1	76.6
RGANet [41]	TIFS23	95.5	89.8	-	-
DPM-SPT [43]	AAAI24	95.5	89.4	91.1	82.4
SCAT [46]	TII24	95.1	88.0	89.3	79.8
OAFR [47]	TMM24	95.3	89.4	90.5	82.1
OAT [20]	TIP24	95.7	89.9	91.2	82.3
FCFormer [27]	TMM24	95.0	89.6	90.6	82.2
MAHATMA [21]	TCSVT25	95.2	88.2	91.2	81.2
HGTDR [48]	TCSVT25	95.6	89.8	91.2	**83.3**
UD-Gaussian [44]	TIP26	**95.9**	89.8	**91.4**	82.7
**DFLNet(Ours)**		**95.9**	**90.2**	91.3	82.7

**Table 3:** Quantitative comparison with state-of-the-art methods on Partial-REID (%) and Partial-iLIDS (%). Bold indicates the best results, while underlining indicates sub-optimal results.

Methods	Reference	Partial-REID		Partial-iLIDS	
		R-1	R-3	R-1	R-3
DSR [33]	CVPR18	50.7	70.0	58.8	67.2
PGFA [15]	ICCV19	68.8	80.0	69.1	80.9
SORN [45]	TCSVT20	76.7	84.3	**79.8**	86.6
PVPM [49]	CVPR20	78.3	87.7	-	-
PFD [16]	AAAI22	67.6	74.3	69.7	80.7
MSHA-Net [50]	TNNLS22	81.3	87.7	73.6	85.4
QPM [14]	TMM22	81.7	88.0	77.3	85.7
AdaSP [51]	CVPR23	74.8	83.2	51.0	58.0
SCAT [46]	TII24	76.3	85.3	73.9	86.6
OAFR [47]	TMM24	**84.3**	-	-	-
**DFLNet(Ours)**		82.3	**89.0**	79.0	**87.4**

**Table 4:** Ablation study of each component on Occluded-DukeMTMC (%). Bold indicates the best results.

Methods	SLO		TDC	DFF	Occluded-Duke	
	S1	S2			Rank-1	mAP
Baseline					64.4	57.3
w/S1	✓				71.4	60.7
w/S2		✓			70.3	60.3
w/SLO	✓	✓			73.0	61.9
w/TDC			✓		68.4	60.9
w/o DFF	✓	✓	✓		74.9	63.1
**Ours**	✓	✓	✓	✓	**76.0**	**63.9**

**Table 5:** Different occlusion simulation methods based on ViT and the generalization ability of the SLO strategy across various models were compared on the Occluded-DukeMTMC (%). Bold indicates the best results, red text represents the performance gain.

Occluded-DukeMTMC			
Backbone	Methods	Rank-1	mAP
ViT	NFDM [25]	67.1	55.9
	CutMix [52]	67.3	60.8
	OIA [27]	71.3	60.9
	PKOS [47]	68.5	57.7
ViT	SLO (Ours)	**73.0**	**61.9**
ViT	TransReID [18]	66.4	59.2
	TransReID + SLO	71.1 (+4.7)	62.8 (+3.6)
	DPM [19]	71.4	61.8
	DPM + SLO	74.2 (+2.8)	63.1 (+1.3)

**Table 6:** Ablation study of DCL on Occluded-DukeMTMC (%). MCT is the extended multiple class tokens, COS represents cosine dissimilarity loss, and EU represents euclidean distance loss. Bold indicates the best results.

Methods	MC	COS	EU	Occluded-DukeMTMC	
				Rank-1	mAP
Baseline				64.4	57.3
w/MCT	✓			67.6	59.3
w/COS	✓	✓		67.9	60.5
w/EU	✓		✓	68.2	60.1
w/TDC	✓	✓	✓	**68.4**	**60.9**

**Table 7:** Comparison with other fusion strategies on Occluded-DukeMTMC (%). Bold indicates the best results.

Methods	Occluded-DukeMTMC	
	Rank-1	mAP
Direct concatenation	74.8	62.7
Weighted Average	74.5	62.9
Ours	**76.0**	**63.9**

**Table 8:** Complexity analysis and performance comparison of the baseline and proposed methods.

Method	Parameters	FLOPs	FPS	Rank-1	mAP
Baseline	92.744M	22.530G	287.9 (sample/s)	64.4	57.3
DFLNet	92.744M	23.481G	275.1 (sample/s)	76.0	63.9

## Data Availability

Data from this study will be made available upon request.
